# Dynamics and Correlation of Serum Cortisol and Corticosterone under Different Physiological or Stressful Conditions in Mice

**DOI:** 10.1371/journal.pone.0117503

**Published:** 2015-02-20

**Authors:** Shuai Gong, Yi-Long Miao, Guang-Zhong Jiao, Ming-Ju Sun, Hong Li, Juan Lin, Ming-Jiu Luo, Jing-He Tan

**Affiliations:** College of Animal Science and Veterinary Medicine, Shandong Agricultural University, Tai-an City, 271018, P. R. China; John Hopkins University School of Medicine, UNITED STATES

## Abstract

Although plasma corticosterone is considered the main glucocorticoid involved in regulation of stress responses in rodents, the presence of plasma cortisol and whether its level can be used as an indicator for rodent activation of stress remain to be determined. In this study, effects of estrous cycle stage, circadian rhythm, and acute and chronic (repeated or unpredictable) stressors of various severities on dynamics and correlation of serum cortisol and corticosterone were examined in mice. A strong (r = 0.6–0.85) correlation between serum cortisol and corticosterone was observed throughout the estrous cycle, all day long, and during acute or repeated restraints, chronic unpredictable stress and acute forced swimming or heat stress. Both hormones increased to the highest level on day 1 of repeated-restraint or unpredictable stresses, but after that, whereas the concentration of cortisol did not change, that of corticosterone showed different dynamics. Thus, whereas corticosterone declined dramatically during repeated restraints, it remained at the high level during unpredictable stress. During forced swimming or heat stress, whereas cortisol increased to the highest level within 3 min., corticosterone did not reach maximum until 40 min. of stress. Analysis with HPLC and HPLC-MS further confirmed the presence of cortisol in mouse serum. Taken together, results (i) confirmed the presence of cortisol in mouse serum and (ii) suggested that mouse serum cortisol and corticosterone are closely correlated in dynamics under different physiological or stressful conditions, but, whereas corticosterone was a more adaptation-related biomarker than cortisol during chronic stress, cortisol was a quicker responder than corticosterone during severe acute stress.

## Introduction

It is known that stress enhances the activity of the hypothalamus-pituitary-adrenal (HPA) axis and results in increased secretion of corticosteroids from the adrenal cortex. Cortisol and corticosterone are thus often used as biomarkers for stress and depressive disorders. Although corticosterone is considered the main glucocorticoid involved in regulation of stress responses in rodents, researchers often choose to detect cortisol for stress indicators in consideration of convenience and kits availability. In fact, several studies have observed increased cortisol in plasma and adrenal glands of mice following stress [1–4], and many studies have even used cortisol as the index for stress activation in both mice [5–8] and rats [9–11]. Furthermore, it has been shown that cortisol exhibits much higher glucocorticoid potency than corticosterone [12]. However, whether cortisol is indeed present in laboratory rodents remains to be determined carefully in a special study using methods with greater specificity.

Studies in rabbits indicated that the ratio of cortisol to corticosterone might be influenced by some physiological conditions. For example, representation of cortisol was observed to be favored in fetal stages of development [13], after stimulation of adrenal secretion with adrenocorticotropin [14–17] as well as in vitro depending on concentration of some precursors needed for hormone biosynthesis [18]. In spite of the fact that corticosterone is the main glucocorticoid in plasma, prevalence of cortisol over corticosterone was revealed in some organs such as kidneys, spleen, heart and brain [19]. Furthermore, in wild species, both cortisol and corticosterone are formed in a species-specific extent and may vary in ratio according to the demands [20,21].

Taken together, the above review suggests that the representativeness of cortisol and corticosterone in rodents may be different under different physiological or stressful conditions. Thus, there is an urgent need for research on the dynamics and correlation of cortisol and corticosterone under different physiological or stressful conditions. In this study, effects of estrous cycle stage, circadian rhythm, and acute and chronic (repeated or unpredictable) stressors of various severities on dynamics and correlation of plasma cortisol and corticosterone were examined in mice.

## Materials and Methods

### Ethics Statement

Mouse care and use were conducted exactly in accordance with the guidelines and approved by the Animal Research Committee of the Shandong Agricultural University, P. R. China (Permit number: 20010510). According to the guidelines of the committee, the animal handling staff (including each post-doc, doctoral or masters student) must be trained before using animals. Mice must be housed in a temperature-controlled room with proper darkness-light cycles, fed with a regular diet, and maintained under the care of the Experimental Animal Center, Shandong Agricultural University College of Animal Science and Vet Medicine. In the present study, mice were sacrificed by decapitation. The only procedure performed on the dead animals was the collection of trunk blood.

Unless otherwise specified, all chemicals and reagents used in the present study were purchased from Sigma Chemical Co. (St. Louis, MO, USA).

### Mice

Mice of the Kunming strain were used at the age of 6–8 weeks. The mice were kept in a room with 14h/10h light-dark cycles, the dark starting from 20:00 pm. The stage of the estrous cycle in female mice was determined by observing vaginal appearance [22] between 19:30 and 20:00 pm on each experimental day. Mice at different stages of the estrous cycle were then sacrificed to collect blood. Immediately following blood collection, the mice were examined again by observing vaginal lavage smears [23] to confirm their estrous cycle stages. Only blood collected from mice at the desired stage of the estrous cycle was used for hormone assays.

### Procedures for restraint stress

Restraint of small animals is an experimental procedure developed for studying psychogenic stress [24,25]. According to Golub et al. [26], psychogenic implies that no invasive physical procedure or tissue trauma is involved but, rather, that the stress response is initiated in the brain by the psychological distress of being unable to move freely. To meet these requirements, we have established a new restraint system in which an individual mouse was put in a micro-cage, constructed by the authors, which was placed in an ordinary home cage. The oblong micro-cage was made of a steel-wire screen and measured 10 cm in width and 2 cm in height. The micro-cage offered the same photoperiod and controlled temperature as in the home cage for the unstressed animals. While in the micro-cage, mice could move back and forth to some extent and could take food and water freely, although they could not turn around. Observations in our laboratory showed that the average intake of food and water did not differ significantly between unstressed control mice and mice restrained for different times using our restraint system (data not shown). Thus, mice restrained in this system did not suffer from any physical suppression or pain. For acute restraint treatment, mice were restrained uninterruptedly for up to 48 h starting from 15:00. For repeated restraint treatment, each restraint session lasted for 8 h (from 8:00 am to 16:00 pm) and one session was conducted daily for up to 23 days. Control mice remained in their home cages during the time treated mice were stressed.

### Procedures for forced swimming stress

For forced swimming testing, animals were forced to swim for different times in a rectangular plastic tank (45×35×18 cm) containing 15 cm deep water. The water temperature was maintained at approximately 23°C. After swimming, the mice were toweled dry before being sacrificed for blood collection.

### Procedures for unpredictable stress

For unpredictable stress treatment, mice were exposed to different stressors for two rounds of 4 days. Stressors used on different days were as follows: Day 1, 2-h heat stress in oven at 42°C; Day 2, 2-h shaker stress (160 rpm); Day 3, 2-h forced swimming at 23°C; and Day 4, 8-h restraint stress. Whereas the stressors lasting for 2 h were administered from 14:00 to 16:00 pm, the 8-h restraint took place from 8:00 am to 16:00 pm as described above.

### Preparation of blood serum

In the present study, mice were always sacrificed at 15:00–16:00 pm for blood collection except for the experiment on the effect of the estrous cycle stage in which mice were always killed at 19:30–20:00 pm and for the experiment on the effect of circadian rhythm in which animals were killed at different times of day. Mice were decapitated rapidly at the end of the stress period, and trunk blood (about 1 ml) was collected into ice-cooled centrifugal tubes and centrifuged (1700 ×g, 10 min, 4°C) to separate serum. The serum collected was divided into two parts; one for assay of cortisol and the other for assay of corticosterone. The serum samples were stored at −80°C until hormone assay.

### Hormone assays

Radioimmunoassay for cortisol was conducted by the Central Hospital of Tai-An City using commercial kits from 3V Biomedical Techniques Co. Ltd., Weifang, China. The kit measures total cortisol in serum including the cortisol combined with corticosteroid-binding globulin (CBG). The minimum level of detection for assays of cortisol was 0.15 ng/ml. The intra- and inter-assay coefficients of variation were <10% and <10%. The cross reactivity of the cortisol RIA kit for corticosterone is 0.11% (tested at the 50% binding). To further evaluate the cross-reactivity of the cortisol RIA with corticosterone, corticosterone and cortisol standards or their mixtures at different concentrations were subjected to the radioimmunoassay kit.

Corticosterone concentrations were measured with an ELISA kit purchased from Arbor Assays Company (Catalog Number K014-H1). The minimum level of detection of the kit was 16.9 pg/ml corticosterone. The kit measures total corticosterone in serum including the corticosterone combined with corticosteroid-binding globulin (CBG). The cross reactivity of the corticosterone kit for cortisol is 0.38% (tested at the 50% binding). Briefly, 50 μl of standards or samples were added in duplicate to wells of the micro-titer plate. 75-μl of assay buffer was added to the non-specific binding (NSB) wells and 50 μl of assay buffer was added to wells to act as maximum binding wells. Then, 25 μl of the DetectX Corticosterone Conjugate and 25 μl of the DetectX Corticosterone Antibody (except the NSB wells) were added to each well and the titer plate was shaken for 1 h at room temperature. After the plate was washed using the wash solution and blot dried by hitting plate onto paper towels, 100 μl of the TMB Substrate were added to each well and incubated for 30 min at room temperature. The optical density (O.D.) of corticosterone was read at 450 nm wavelength using a plate reader within 15 min after the reaction was terminated by adding 50 μl of the Stop Solution. The concentration of corticosterone was calculated according to the standard curves.

### HPLC and ESIMS analysis of glucocorticoids in mouse serum

To measure serum glucocorticoids, a liquid-liquid extraction was performed by adding 1.6 ml ether to 0.8 ml of serum and vortexing for 2 min. After 10 min of rotating at 1200×g, the sample was centrifuged at 3800×g for 10 min at 4°C. The organic layer was collected and transferred to a 15 ml tube. This liquid-liquid extraction was repeated by adding another 1.6 ml ether to the remaining serum material. The organic phase was dried under a gentle stream of nitrogen at a temperature of 40°C and reconstituted in 2 ml methanol for HPLC analyses. Qualitative detection of the glucocorticoids in serum of mice was performed by reversed phase high performance liquid chromatography (HPLC) and electrospray ionization mass spectrometry (ESIMS). The HPLC was conducted on an Agilent 1260 series instrument (Agilent Technologies, Waldbron, Germany) equipped with an in-line degasser, quaternary pump, autosampler and a diode array and multiple wavelength detector, using a 5-μm YMC-Pack Pro C18 (250 mm×4.6 mm i.d.) column for separation, with acquisition set at 242 nm for reference substances and serum samples of mice. The ESIMS was carried out on a Thermo Finnigan MSQ 10275. Mass and MS/MS spectra were achieved by ESI in positive ion mode. The electrospray voltage was set at 4.2 kV, the capillary temperature at 300°C and vaporizer temperature at 350°C. The sheath and auxiliary gas are nitrogen and their pressures were set to 40 and 5 arbitrary units. The mobile phases for HPLC consisted of water: methyl alcohol (40: 60% v/v) at a flow rate 1 ml min^-1^. The mobile phases for ESIMS consisted of 2 mM ammonium acetate: methyl alcohol (40: 60% v/v) at a flow rate 0.7 ml min-1. The injection volume was 20 μl and the column temperature was set at 35°C. Peaks were identified based on the retention time of the standards and confirmed by comparison of the wavelength scan spectra (set between 210 nm and 400 nm).

### Data analysis

At least three replicates were performed for each treatment. Percentage data were arc sine transformed and analyzed with ANOVA; a Duncan multiple comparison test was used to locate differences. The Statistical Package for Social Science software (version 11.5; SPSS, Inc.) was used. Data are expressed as the mean ± SEM, with P<0.05 considered to be statistically significant. Pearson correlation coefficient test was performed to determine the correlation between cortisol and corticosterone under different circumstances, with P<0.05 considered to be statistically significant. All values were checked for Gaussian distribution by K-S (Kolmogorov-Smirnov) in SPSS before further analysis.

## Results

### Effects of the estrous cycle stage on the dynamics and correlation of serum cortisol and corticosterone

**Fig 1:**
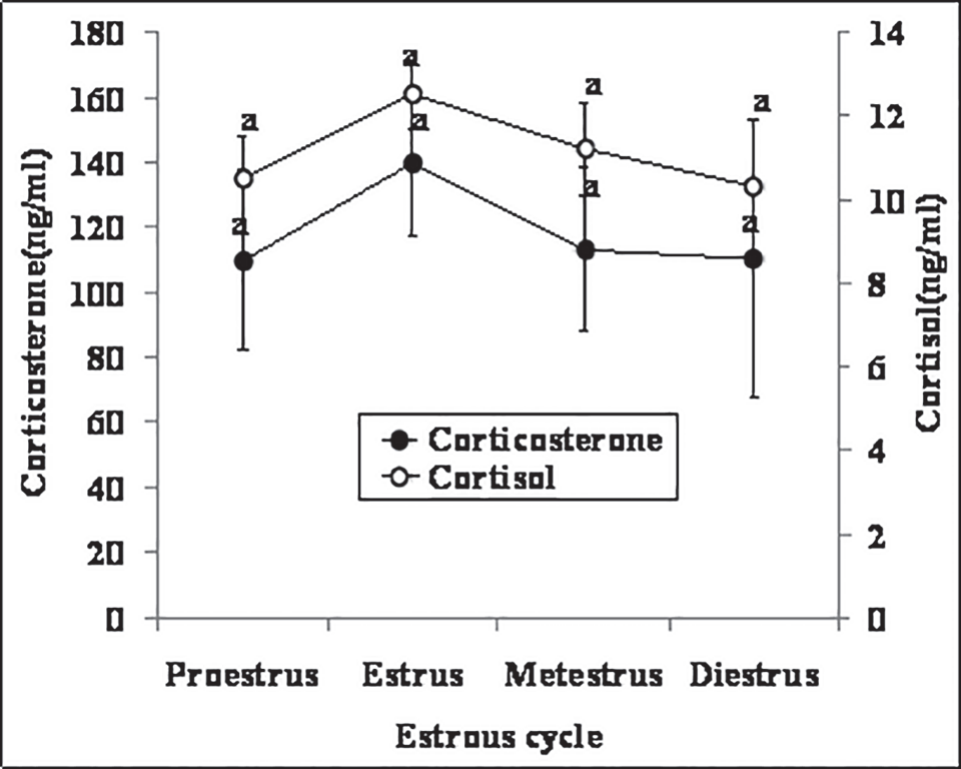
Concentrations of serum cortisol and corticosterone in female mice (n = 6) at different stages of the estrous cycle. Values without a common letter differ (P < 0.05) within cortisol or corticosterone groups.

Between 19:30 and 20:00 on each experimental day, female mice were examined for the stage of the estrous cycle by observing vaginal appearance. Mice at different stages of the estrous cycle were then sacrificed to collect blood. Immediately following blood collection, the mice were examined again by observing vaginal lavage smears to confirm their estrous cycle stages. No significant changes were observed in either cortisol or corticosterone concentration throughout the estrous cycle (Fig. 1). However, contents of both hormones were higher at estrus than at other stages although the difference did not reach statistical significance. A correlation analysis showed that concentration of cortisol was strongly correlated with that of corticosterone throughout the estrous cycle (r = 0.786, P <0.01).

### Effects of the circadian rhythm on the dynamics and correlation of serum cortisol and corticosterone

**Fig 2:**
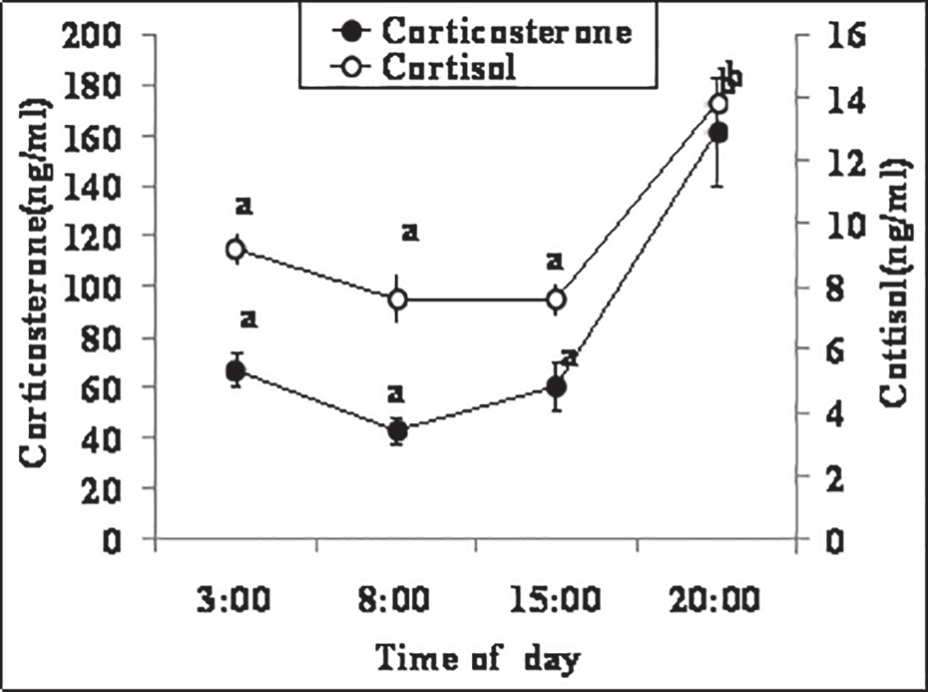
Concentrations of serum corticosterone and cortisol in male mice (n = 6) at different times of day. Values without a common letter differ (P < 0.05) within cortisol or corticosterone groups.

Male mice were sacrificed at different times of day to collect blood for assay of cortisol and corticosterone. Concentrations of both cortisol and corticosterone remained constant from 3:00 in the early morning to 15:00 in the afternoon, but they went up significantly at 20:00 in the evening (Fig. 2). A correlation test showed a strong correlation between the two hormones at the four time points tested (r = 0.846, P <0.01).

### Effects of acute restraint stress on the dynamics and correlation of serum cortisol and corticosterone

**Fig 3:**
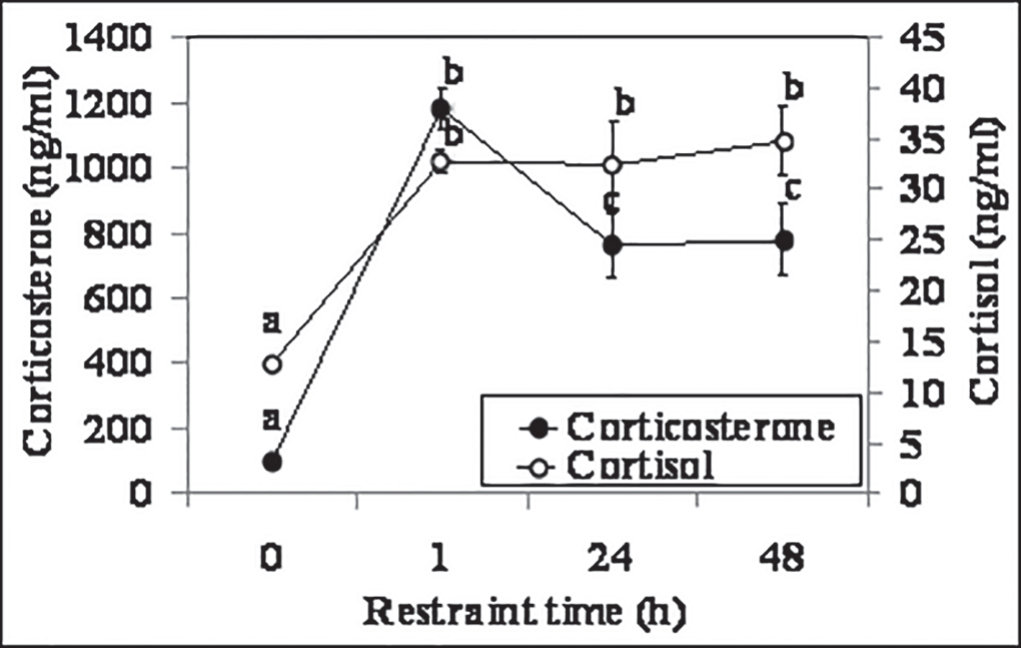
Concentrations of serum cortisol and corticosterone in female mice (n = 6) at different times (h) of acute restraint stress. Values without a common letter differ (P < 0.05) within cortisol or corticosterone groups.

Female mice were restrained for different times before being sacrificed to collect blood for hormone assays. Concentrations of both cortisol and corticosterone went up to the highest level within 1 h of restraint stress (Fig. 3). However, whereas the level of cortisol stayed high up to 48 h of restraint, the level of corticosterone decreased significantly at 24 and 48 h of restraint. Within 1 h after the animal was released from a 48-h restraint, the level of serum cortisol and corticosterone returned to the level observed in unstressed control (0 h) mice (data not shown). A correlation test showed a strong correlation between the two hormones at the four time points tested (r = 0.847, P <0.01). Results suggested that the dynamics of serum corticosterone could reflect the course of animal adaptation to a stressor better than could the dynamics of the serum cortisol.

### Effects of chronic restraint stress on the dynamics and correlation of serum cortisol and corticosterone

**Fig 4:**
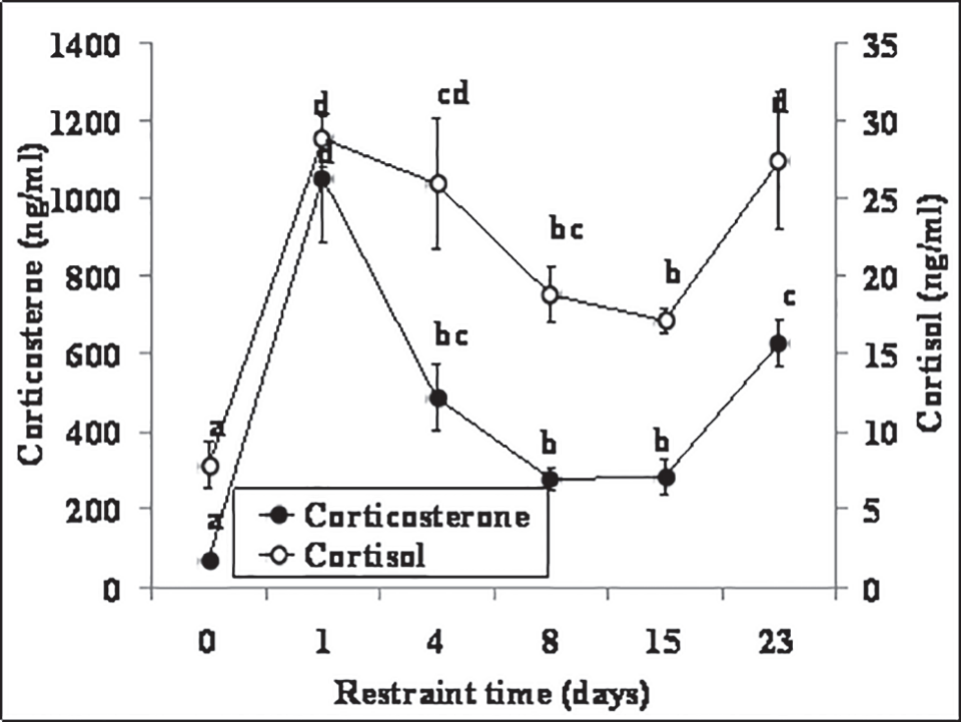
Concentrations of serum cortisol and corticosterone in female mice (n = 6) on different days of chronic restraint stress. Values without a common letter differ (P < 0.05) within cortisol or corticosterone groups.

Female mice were repeatedly restrained for different days before being sacrificed to collect blood for hormone assays. Concentrations of both cortisol and corticosterone went up to the highest level on day 1 of restraint (Fig. 4). However, although both hormones declined afterwards, the descent rate of corticosterone was faster than that of cortisol. On day 4 of restraint, for example, whereas the cortisol concentration did not differ significantly from that on day 1, the corticosterone concentration decreased significantly from that on day 1. On day 8 of restraint, whereas the cortisol concentration was only 1.5-fold less than on day 1 of restraint (19 ng/ml versus 29 ng/ml), the corticosterone concentration was 3.8-fold less than that on day 1 (280 ng/ml versus 1050 ng/ml). A strong correlation was observed between the two hormones on different days of restraint (r = 0.757, P<0.01). Again, results suggested that corticosterone decreased more remarkably than cortisol as the mice adapted to the restraint stress.

### A comparison on cortisol and corticosterone dynamics between repeated restraint and unpredictable stressors

**Fig 5:**
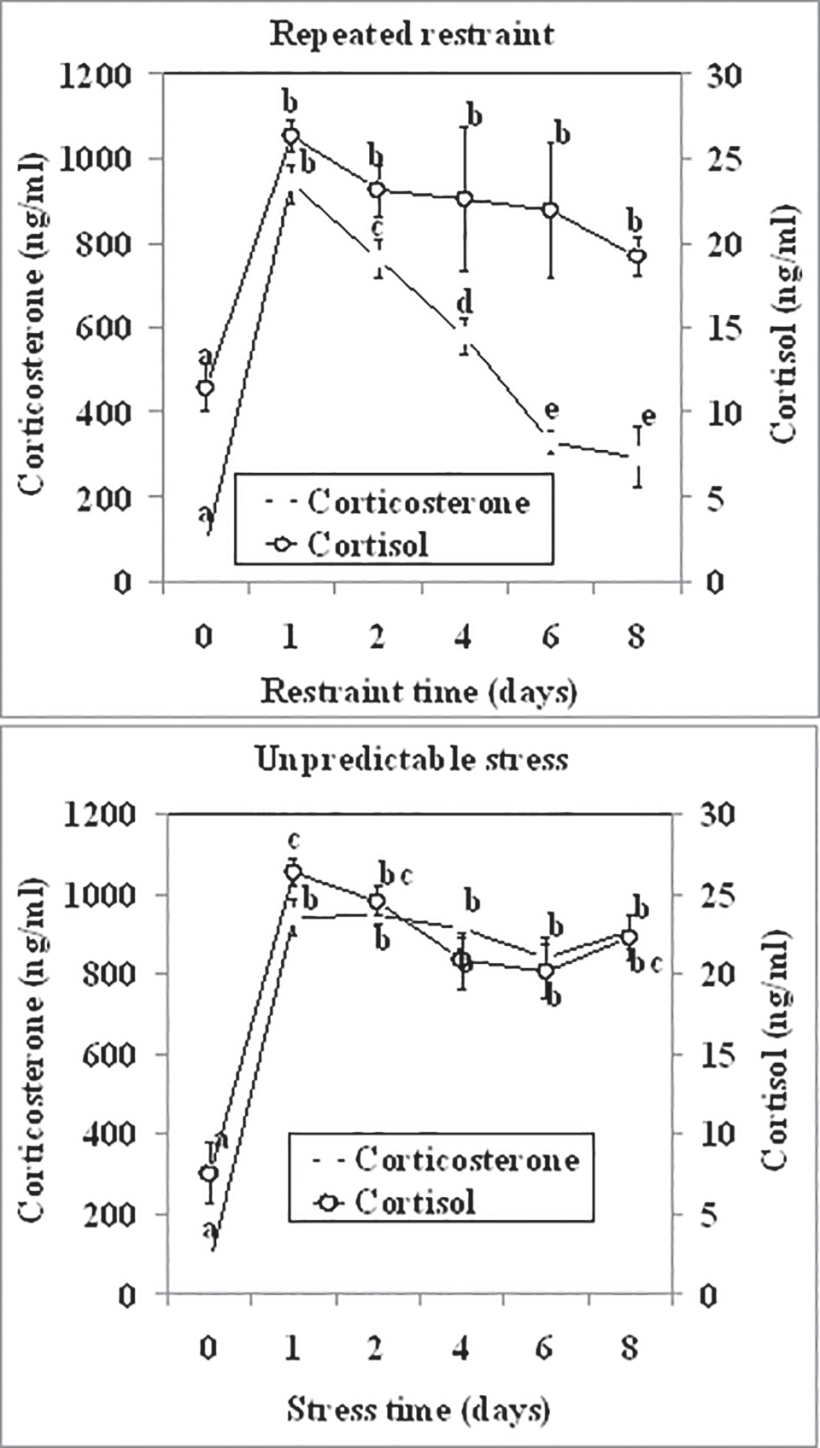
Concentrations of serum cortisol and corticosterone in male mice (n = 6) on different days of repeated restraint stress or unpredictable stress. Values without a common letter differ (P < 0.05) within cortisol or corticosterone groups.

To verify our above conclusion that the dynamics of corticosterone was more adaptation-reflective than that of cortisol, cortisol and corticosterone dynamics was compared between the repeated restraint stress that is known to cause animal adaptations and the unpredictable stress that is supposed not to induce animal adaptations. Male mice were exposed to repeated restraints or unpredictable stressors for different days before being sacrificed to collect blood for hormone assays. Both hormones went up to the highest level on day 1 of both repeated restraint and unpredictable stresses (Fig. 5). After that, however, whereas the concentration of cortisol remained at the high level up to day 8 in both stress systems, the level of corticosterone showed different dynamics between repeated restraint and unpredictable stresses. Thus, whereas corticosterone declined dramatically on each day of repeated restraints, it did not change significantly up to day 8 of the unpredictable stress. Although a strong correlation was observed between the two hormones during both repeated restraint and unpredictable stresses, the correlation coefficient was lower following repeated restraint stress (r = 0.64) than following the unpredictable stress (r = 0.885). Results confirmed that the dynamics of corticosterone could be used as a better maker for animal adaptation to stress than could that of cortisol.

### Rapid effects of forced swimming and heat stress on dynamics and correlation of serum cortisol and corticosterone

**Fig 6:**
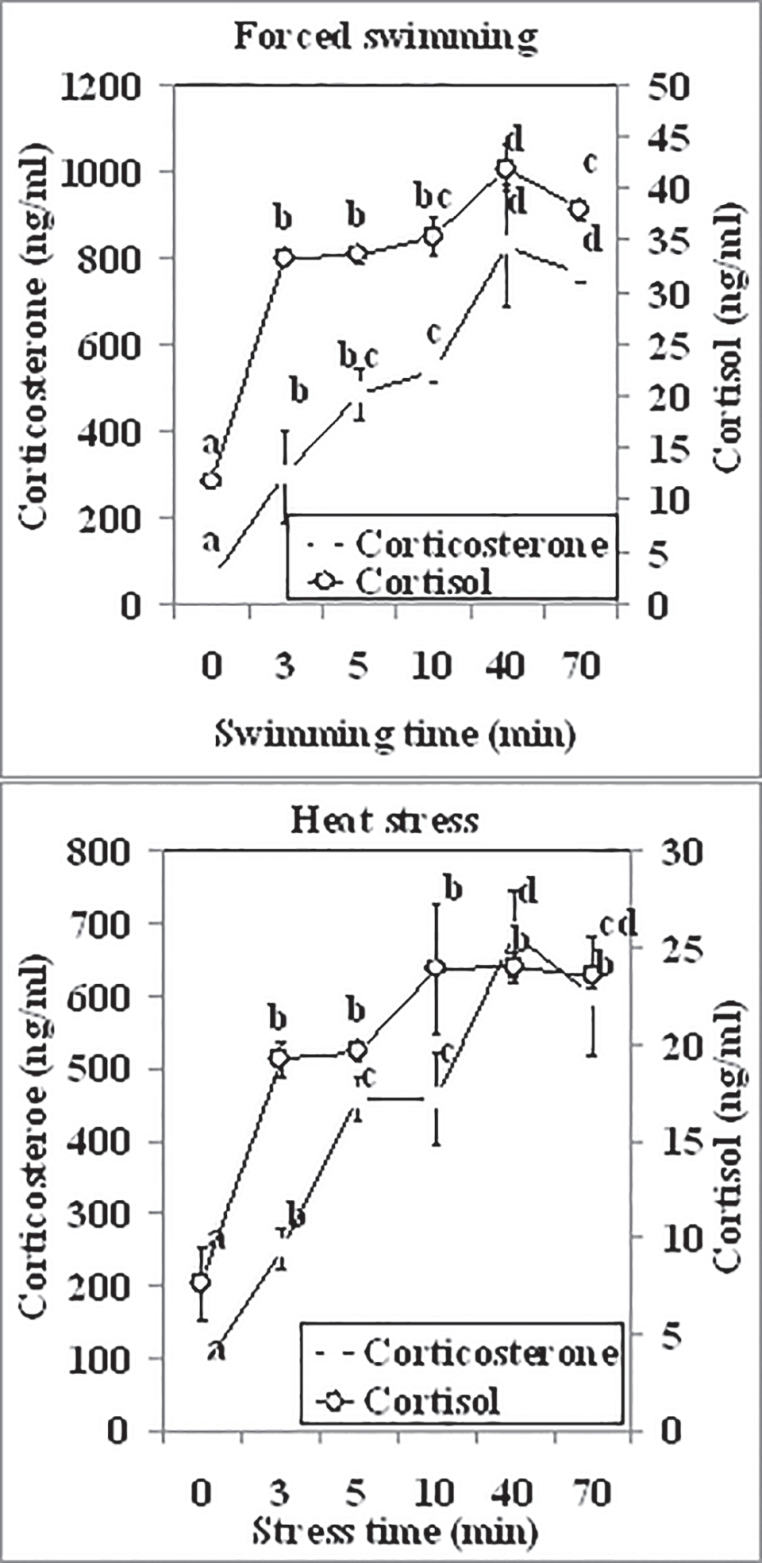
Concentrations of serum cortisol and corticosterone in male mice (n = 6) at different times (min) of forced swimming or heat stress. Values without a common letter differ (P < 0.05) within cortisol or corticosterone groups.

To study the rapid effects of severe stresses on dynamics and correlation of serum cortisol and corticosterone, male mice were exposed to forced swimming or heat stress for different times before being sacrificed to collect blood for hormone assays. During both forced swimming and heat stresses, whereas the concentration of cortisol increased to the highest level within 3 min, the concentration of corticosterone did not reach the highest level until 40 min of the stresses (Fig. 6). A strong correlation was observed between the two hormones during both forced swimming and heat stresses, and the correlation coefficients were 0.594 and 0.767 for forced swimming and heat stresses, respectively. Results suggested that cortisol and corticosterone responded differently to severe stressors with cortisol being a quicker responder than corticosterone.

### Further confirmation of the presence of cortisol in mouse serum

**Fig 7:**
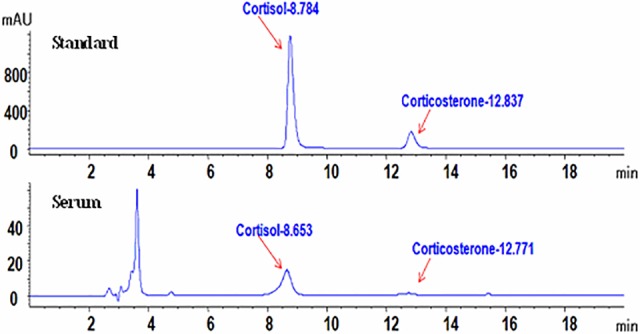
HPLC chromatograms of cortisol and corticosterone in standards and serum from unstressed male mice. Each treatment was repeated 3 times using serum from 10 unstressed mice.

Because of the possibility that the radioimmunoassay used for cortisol measurement in the above experiments might have cross-reactions with corticosterone, two experiments were conducted to verify whether cortisol is indeed present in the mouse blood. First, corticosterone and cortisol standards or their mixtures at different concentrations were subjected to the radioimmunoassay. Results showed that the cortisol concentration detected in the cortisol standard sample containing 10 ng/ml cortisol did not differ from that detected in the mixture of 1000 ng/ml corticosterone and 10 ng/ml cortisol (Table 1). All the cortisol concentrations detected in different samples were lower than the cross-reaction rate labeled on the radioimmunoassay kit (0.11%). Second, qualitative detection of glucocorticoids in serum of male mice was performed by reversed phase HPLC. The HPLC chromatogram (Fig. 7) shows that the retention time of cortisol and corticosterone standards were 8.70±0.05 min and 12.81±0.02 min, respectively. The retention time for cortisol and corticosterone extracted from serum were 8.66±0.01 min and 12.72±0.05 min, respectively. Analysis with HPLC-MS further verified that the molecular weight of cortisol and corticosterone in serum were consistent with that of reference substances (Data not shown).

**Table 1:** Cortisol concentrations detected by radioimmunoassay of corticosterone and cortisol standards or their mixtures at different concentrations.

**Samples containing**		**Cortisol detected (ng/ml)**
**Corticosterone (ng/ml)**	**Cortisol (ng/ml)**	
0	10	9.8±0.26^a^
1000	0	0.6±0.09^b^
1000	10	10.0±0.26^a^
3000	0	1.9±0.23^c^
3000	10	11.8±0.08^d^
9000	0	5.0±0.11^e^
9000	10	15.1±0.20^f^

a-f: Values with a different letter in their superscripts differ (P<0.05). Each treatment was repeated 4 times.

## Discussion

The present results showed that the dynamics of serum cortisol was closely correlated with that of corticosterone under all the physiological or stressful conditions tested (r = 0.6−0.85). Both hormones increased to the highest level on day 1 of repeated-restraint or unpredictable stress, but after day 1, whereas the concentration of cortisol did not change, the level of corticosterone showed different dynamics. Thus, whereas corticosterone declined dramatically during repeated restraints, it did not change significantly during unpredictable stresses. During forced swimming or heat stress, whereas cortisol increased to the highest level within 3 min, corticosterone did not reach maximum until 40 min of stress. Furthermore, the smallest coefficients we observed during forced swimming (r = 0.594) and repeatedly restraint stresses for 8 days (r = 0.64) further verified the different dynamics between cortisol and corticosterone during these two stresses. Taken together, the results suggest that mouse serum cortisol and corticosterone are closely correlated in dynamics under different physiological or stressful conditions, suggesting that both hormones can be interchangeably used as an indicator for stress activation in mice. However, whereas corticosterone is a more adaptation-related biomarker than cortisol during chronic stress, cortisol is a quicker responder than corticosterone during severe acute stress. In guanacos (Lama guanicoe), it has been shown that cortisol and corticosterone exhibit different patterns in the field and in response to acute stressors [21].

Previous studies observed similar dynamics of corticosterone in rodents under the stressful conditions similar to those tested in the present study. For example, Mizobe et al. [27] observed a significant elevation of endogenous corticosterone levels in mice following a 24 h acute restraint stress. Liu et al. [28] reported that serum corticosterone levels in mice exposed to chronic restraint stress for 21 days (242.55 ng/ml) increased significantly compared with those in unstressed control mice (114.45 ng/ml), although the corticosterone level on day 1 of stress was not measured in their study. Wu et al. [29] observed that the plasma corticosterone concentration in mice that had been subjected to chronic unpredictable tress for 30 days was significantly higher than that in control mice, although they did not assay the hormone levels on different days of stress. Ide et al. [30] observed significant elevations in plasma corticosterone in mice subjected to forced-swimming for 6 min per day for five consecutive days. Furthermore, Cavigelli and McClintock [31] found that serum corticosterone in rats exposed to a 5-min exploration-arena test or to a 30-min restraint did not reach the highest level until 40 min after the end of the treatment.

Previous studies also showed cortisol elevations in rodents under the stressful conditions similar to those tested in the present study. For example, a significant elevation in cortisol has been observed in mice after acute restraint stress [5,8]. Plasma cortisol levels were significantly increased in rats subjected to chronic repeated restraint stress for 14 days [10] or exposed to unpredictable stress for 21 days [11]. Furthermore, plasma cortisol contents were significantly elevated in mice following forced swimming test [6]. However, although those studies measured cortisol in laboratory rodents, they did not deal with cortisol in blood samples from a methodological/analytical perspective. Thus, whether cortisol is indeed present in laboratory rodents needs to be carefully determined. The current results demonstrate that the radioimmunoassay we used to detect cortisol had negligible cross-reactions with corticosterone and confirmed unequivocally the presence of cortisol in mouse serum.

Although Champlin [32] found no difference in plasma corticosterone at different stages of the cycle in mice, Nichols and Chevins [33] observed that compared with values recorded at metestrus and diestrus, elevated corticosterone levels were recorded throughout the day and night of pro-estrus and only subsided late in the day of estrus. Because the effect of the estrous cycle stage on hormone secretion may be influenced by the high variability of the mouse estrous cycle [34] and by the circadian rhythm [35], the present study conducted blood collection at a fixed time (19:30–20:00 in the evening) and determined stages of the estrous cycle twice, immediately before and after the blood collection. Even so, we did not observe any significant change in either cortisol or corticosterone concentration throughout the estrous cycle, although contents of both hormones were slightly higher at estrus than at other stages. It is known that the estrous period is characterized by high estrogen secretion from pre-ovulatory follicles [36]. Both in vivo [37,38] and in vitro studies [39] showed that estradiol enhanced the production of corticosterone by adrenal glands in rats.

The present results indicated that while concentrations of both cortisol and corticosterone remained constant from 3:00 in the early morning to 15:00 in the afternoon, they elevated significantly at 20:00 in the evening. One striking feature of the regulation of glucocorticoids is a diurnal release pattern, with peak levels linked to the start of the daily activity phase [40]. In rodents, for example, there are no endogenous corticosterone pulses during the early light phase but hourly pulses of corticosterone secretion are observed during the early dark phase [41]. More specifically, Kim et al. [35] observed in mice that serum corticosterone concentrations were significantly lower at 8:00–11:00 h than at 15:00–18:00 h. A distinct diurnal rhythm in plasma corticosterone was evident in male and female rats, with the average peak time ranging from 21 min before to 70 min after lights off [42].

In summary, although plasma corticosterone is considered the main glucocorticoid involved in regulation of stress responses in rodents, the presence of plasma cortisol and whether its level can be used as an indicator of stress has not been determined in rodents before. Because previous studies indicated that the ratio of cortisol to corticosterone might be influenced by physiologic conditions, developmental stages and organ differences, we observed the effects of estrous cycle stage, circadian rhythm, and acute and chronic (repeated or unpredictable) stressors of various severities on dynamics and correlation of plasma cortisol and corticosterone in mice. Results (i) confirmed the presence of cortisol in mouse serum and (ii) suggested that mouse serum cortisol and corticosterone were closely correlated in dynamics under different physiological or stressful conditions, but whereas corticosterone was a more adaptation-related biomarker than cortisol during chronic stress, cortisol was a quicker responder than corticosterone during severe acute stress. Although the mechanisms for the different dynamics between the two hormones under different stressful conditions are not clear, a recent study by Taves et al. [43] has provided important evidence. They measured high concentrations of cortisol in thymus, bone marrow, and heart of neonatal mice and demonstrated widespread local regulation of glucocorticoid levels during mouse development. It is known that stress can activate not only the HPA axis but also other pathways in the neuroendocrine system. The activation of different neuroendocrine pathways by different stressors may differentially affect the local regulation of glucocorticoid levels in different organs, leading to a different dynamics between cortisol and corticosterone under different stressful conditions. Therefore, the present results must be taken into account when choosing a biomarker for activation of different stresses.
